# Supplementary material for: Foreshock properties illuminate nucleation processes of slow and fast laboratory earthquakes
Source: Nat Commun. 2023 Jun 29;14:3859. doi: 10.1038/s41467-023-39399-0 (PMC10310758; doi:10.1038/s41467-023-39399-0)
Supplement: Supplementary file 1 — Supplementary Information [file 41467_2023_39399_MOESM1_ESM.docx]

­­­

Foreshock properties illuminate nucleation processes of slow and fast laboratory earthquakes

David C. Bolton^1,4*^, Chris Marone^2,3^, Demian Saffer^4^, and Daniel T. Trugman^5^

^1^ Bureau of Economic Geology, Jackson School of Geosciences, University of Texas, Austin, TX.

^2^Department of Geosciences, Pennsylvania State University, University Park, Pennsylvania.

^3^Departimento di Scienze della Terra, La Sapienza Universita di Roma, Italy.

^4^ University of Texas Institute for Geophysics, Jackson School of Geosciences, University of Texas, Austin, TX.

^5^Nevada Seismological Laboratory, University of Nevada, Reno, Nevada


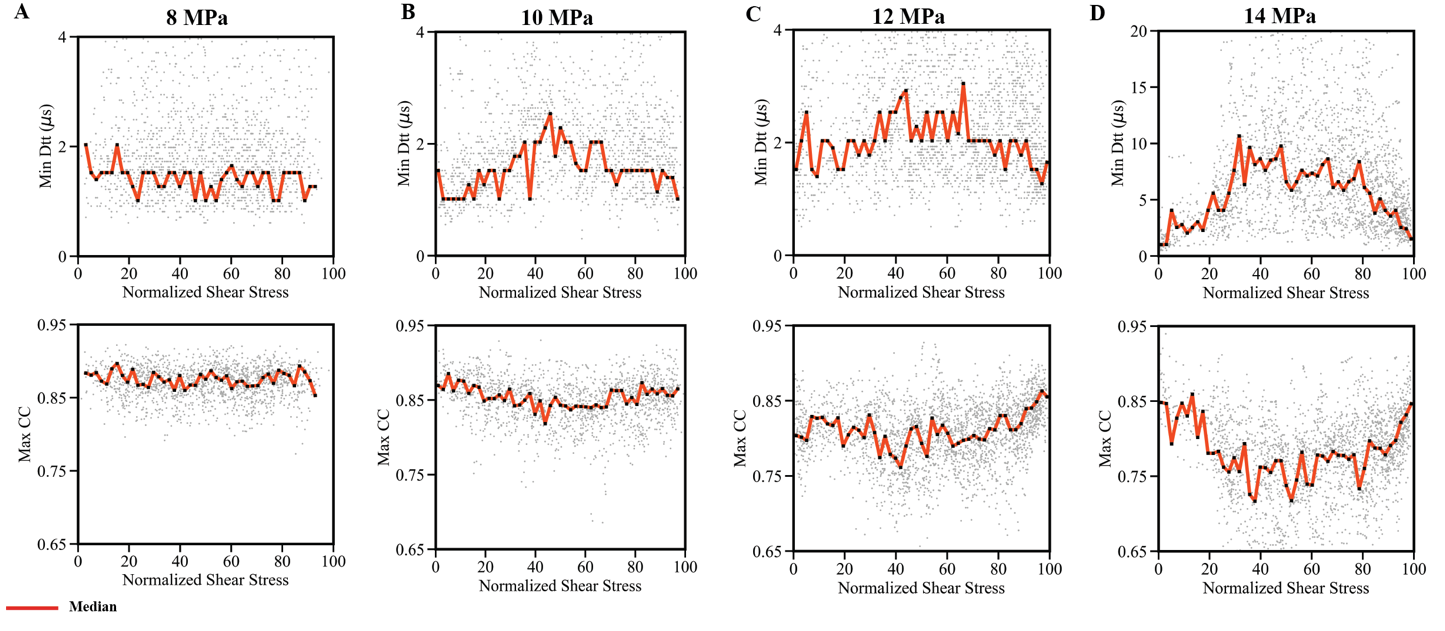


Figure S1. Evolution of waveform similarity and differential travel times throughout the seismic cycle. A-D. Minimum differential travel-times (DTT; top panel) and waveform similarity (bottom panel) as a function of normalized shear stress for slow (A-B) and fast (C-D) stick-slip instabilities. Grey dots represent raw measurements derived from moving window approach and red line represents moving median of the grey points. DTT and waveform similarity remain constant throughout the seismic cycle for the slowest stick-slip events at 8 MPa. Slip events at 10 and 12 MPa show a modest increase followed by a slight decrease in DTT before failure. Waveform similarity tracks DTT and decreases slightly before increasing prior to failure. The fastest events at 14 MPa show a similar behavior as those at 10 and 12 MPa, but much stronger. That is, DTT increase during the initial stages of the seismic cycle (< 40% of peak stress), remain constant, and finally decrease once the fault surpasses ~ 80% of its peak stress. Similarly, waveform similarity decreases during the initial stages of the seismic cycle, remains constant, and finally increases immediately before failure.


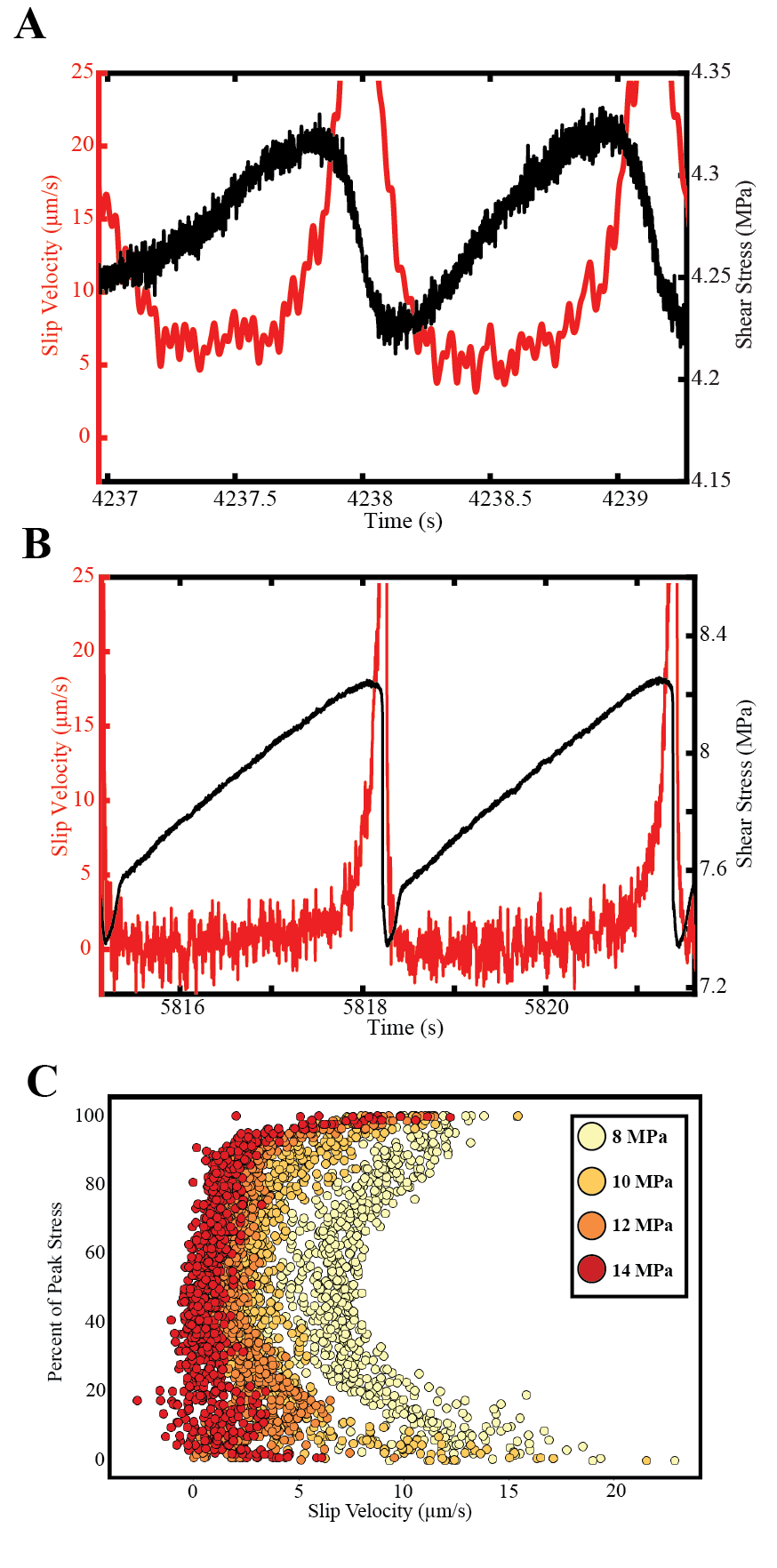


**Figure S2** **Co-evolution of shear stress and slip velocity for slow and fast laboratory earthquakes.** **A-B:** Fault slip velocity and shear stress as a function of time for slow stick-slip events (A; 8 MPa) and fast stick-slip events (B;14 MPa). For slow slip events, the fault reaches a minimum slip rate of ~ 5 μm/s prior to accelerating during the latter stages of the seismic cycle. In contrast, during the fast stick-slip events (B) the fault locks up and reaches a minimum slip rate of ~ 0 μm/s. **C.** Normalized shear stress as a function of slip velocity for 5 seismic cycles; data points are color coded according to their respective normal stress. Note, the fault completely locks up during the inter-seismic period for the fast stick-slip events at 12-14 MPa. At 8 MPa the fault never locks up and continues to slip throughout the entire inter-seismic period.

**Figure S3 Waveform clusters and AE families for slow laboratory earthquakes.** **Top row:** Shear stress versus time for 5 different slow slip cycles at 8 MPa. **Middle Row:** Dendrogram derived from a Hierarchical Agglomerative Clustering (HAC) algorithm. For each slip cycle, we cross-correlate all event pairs during the inter-seismic period and use a distance matrix of 1-C_i,j_, where C_i,j_ represents the median cross-correlation coefficient between events i and j . Colors are used to denote different clusters and black solid line represents distance threshold used to define clusters. The 0.4 distance threshold indicates that each cluster is composed of waveforms with similarity values between 0.6-1. The distance threshold has a strong influence on the number of clusters identified but increasing the threshold above 0.4 is not ideal as this would allow dissimilar AEs (i.e., cross-correlation coefficients < 0.5) to be a part of the same cluster. Each slow slip cycle contains between 23-36 families of AEs. **Bottom Row**: Number of AE families as a function of position in the seismic cycle. The data do not show a systematic relationship between the number of families and position within the seismic cycle.

**Figure S4 Waveform clusters and AE families for fast laboratory earthquakes.** **Top row:** Shear stress versus time for 5 different fast slip cycles at 14 MPa. **Middle Row:** Dendrogram derived from a Hierarchical Agglomerative Clustering (HAC) algorithm. Data are derived in a similar manner as those in Figure S3. Colors are used to denote different clusters and black solid line represents distance threshold used to define clusters. The data show that each slip cycle has between 12-15 different families of AEs. **Bottom Row**: Number of families as a function of position in the seismic cycle. Aside from the slip cycle between 5722-5724 s, the data show a modest increase in the number of AE families as failure approaches.
